# (*E*)-4-Chloro-*N*′-[1-(4-hydroxy­phenyl)­ethylidene]benzohydrazide

**DOI:** 10.1107/S1600536808027001

**Published:** 2008-08-30

**Authors:** De-Suo Yang

**Affiliations:** a Department of Chemistry and Chemical Engineering, Baoji University of Arts and Sciences, Baoji 721007, People’s Republic of China

## Abstract

The mol­ecule of the title compound, C_15_H_13_ClN_2_O_2_, displays a *trans* configuration with respect to the C=N double bond. The dihedral angle between the two benzene rings is 15.1 (3)°. A strong intra­molecular O—H⋯N hydrogen bond is observed. In the crystal structure, mol­ecules are linked through inter­molecular N—H⋯O hydrogen bonds, forming chains running along [101].

## Related literature

For bond-length data, see: Allen *et al.* (1987 ▶). For related structures, see: Yang (2007 ▶, 2008*a* ▶,*b* ▶). For general background, see: Bernardo *et al.* (1996 ▶); Musie *et al.* (2001 ▶); Paul *et al.* (2002 ▶).
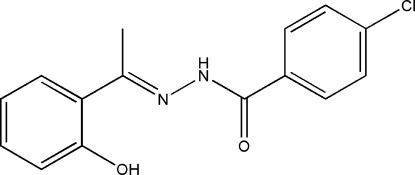


## Experimental

### 

#### Crystal data

C_15_H_13_ClN_2_O_2_
*M* *_r_* = 288.72
Monoclinic, 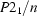

*a* = 7.241 (3) Å
*b* = 23.653 (4) Å
*c* = 8.744 (3) Å
β = 113.682 (3)°
*V* = 1371.5 (8) Å^3^
*Z* = 4
Mo *K*α radiation
μ = 0.28 mm^−1^
*T* = 298 (2) K
0.32 × 0.30 × 0.28 mm

#### Data collection

Bruker SMART CCD area-detector diffractometer
Absorption correction: multi-scan (*SADABS*; Sheldrick, 1996 ▶) *T*_min_ = 0.915, *T*_max_ = 0.925
11286 measured reflections
2961 independent reflections
1543 reflections with *I* > 2σ(*I*)
*R*_int_ = 0.071

#### Refinement

*R*[*F* ^2^ > 2σ(*F* ^2^)] = 0.058
*wR*(*F* ^2^) = 0.143
*S* = 0.99
2961 reflections
186 parameters
1 restraint
H atoms treated by a mixture of independent and constrained refinement
Δρ_max_ = 0.26 e Å^−3^
Δρ_min_ = −0.22 e Å^−3^

### 

Data collection: *SMART* (Bruker, 2002 ▶); cell refinement: *SAINT* (Bruker, 2002 ▶); data reduction: *SAINT*; program(s) used to solve structure: *SHELXS97* (Sheldrick, 2008 ▶); program(s) used to refine structure: *SHELXL97* (Sheldrick, 2008 ▶); molecular graphics: *SHELXTL* (Sheldrick, 2008 ▶); software used to prepare material for publication: *SHELXL97*.

## Supplementary Material

Crystal structure: contains datablocks global, I. DOI: 10.1107/S1600536808027001/ci2658sup1.cif

Structure factors: contains datablocks I. DOI: 10.1107/S1600536808027001/ci2658Isup2.hkl

Additional supplementary materials:  crystallographic information; 3D view; checkCIF report

## Figures and Tables

**Table 1:** Hydrogen-bond geometry (Å, °)

*D*—H⋯*A*	*D*—H	H⋯*A*	*D*⋯*A*	*D*—H⋯*A*
O2—H2⋯N2	0.82	1.80	2.513 (3)	145
N1—H1⋯O1^i^	0.90 (1)	2.074 (11)	2.968 (3)	176 (3)

Symmetry code: (i) 
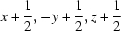
.

**Fig. 1.**
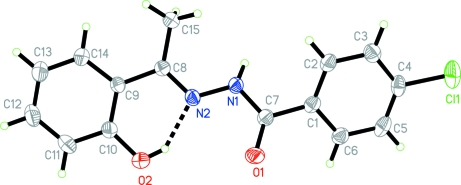
The molecular structure of the title compound, showing the atom-numbering scheme. Displacement ellipsoids are drawn at the 30% probability level.

**Fig. 2.**
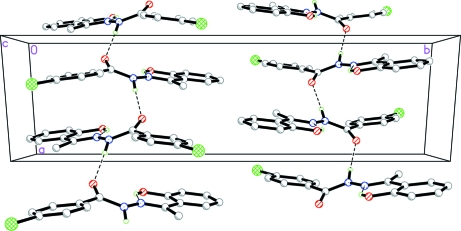
Molecular packing as viewed along the c axis. Hydrogen bonds are shown as dashed lines.

## supplementary crystallographic information

### Comment

Schiff base compounds have been of great interest for a long time. These compounds play an important role in the development of coordination chemistry (Musie *et al.*, 2001; Bernardo *et al.*, 1996; Paul *et al.*, 2002). Recently, we have reported a few Schiff base compounds (Yang, 2007, 2008*a*,*b*). As a further investigation of this work, the crystal structure of the title compound is reported here.

The molecule of the title compound displays a *trans* configuration with respect to the C?N double bond (Fig. 1). The dihedral angle between the two benzene rings is 15.1 (3)°. All the bond lengths are within normal ranges (Allen *et al.*, 1987). The C8═N2 bond length of 1.287 (3) Å conforms to the value for a double bond. The N1—C7 bond length of 1.355 (3) Å is intermediate between a C—N single bond and a C?N double bond, because of conjugation effects in the molecule. There is a strong intramolecular hydrogen bond between the hydroxyl hydrogen and N2.

In the crystal structure, molecules are linked through intermolecular N—H···O hydrogen bonds (Table 1), forming chains running along the [1 0 1] direction (Fig. 2).

### Experimental

1-(2-Hydroxyphenyl)ethanone (0.1 mmol, 13.6 mg) and 4-chlorobenzohydrazide (0.1 mmol, 17.0 mg) were dissolved in MeOH (10 ml). The mixture was stirred at room temperature to give a clear colourless solution. Single crystals of the title compound were obtained by gradual evaporation of the solvent over a period of 12 d at room temperature.

### Refinement

Atom H1 was located in a difference Fourier map and refined isotropically, with N—H distance restrained to 0.90 (1) Å and with a *U*_iso_ value of 0.08 Å^2^. Other H atoms were placed in idealized positions and constrained to ride on their parent atoms, with a O—H distance of 0.82 Å, C—H distances of 0.93–0.96 Å, and with *U*_iso_(H) = 1.2*U*_eq_(C) and 1.5*U*_eq_(O2 and C15).

### Crystal data

**Table:**

C_15_H_13_ClN_2_O_2_	*F*_000_ = 600
*M**_r_* = 288.72	*D*_x_ = 1.398 Mg m^−^^3^
Monoclinic, *P*2_1_/*n*	Mo *K*α radiation λ = 0.71073 Å
Hall symbol: -P 2yn	Cell parameters from 872 reflections
*a* = 7.241 (3) Å	θ = 2.6–24.5º
*b* = 23.653 (4) Å	µ = 0.28 mm^−^^1^
*c* = 8.744 (3) Å	*T* = 298 (2) K
β = 113.682 (3)º	Block, colourless
*V* = 1371.5 (8) Å^3^	0.32 × 0.30 × 0.28 mm
*Z* = 4	

### Data collection

**Table:**

Bruker SMART CCD area-detector diffractometer	2961 independent reflections
Radiation source: fine-focus sealed tube	1543 reflections with *I* > 2σ(*I*)
Monochromator: graphite	*R*_int_ = 0.071
*T* = 298(2) K	θ_max_ = 27.0º
ω scans	θ_min_ = 1.7º
Absorption correction: multi-scan(SADABS; Sheldrick, 1996)	*h* = −9→9
*T*_min_ = 0.916, *T*_max_ = 0.926	*k* = −29→30
11286 measured reflections	*l* = −10→11

### Refinement

**Table:**

Refinement on *F*^2^	Secondary atom site location: difference Fourier map
Least-squares matrix: full	Hydrogen site location: inferred from neighbouring sites
*R*[*F*^2^ > 2σ(*F*^2^)] = 0.058	H atoms treated by a mixture of independent and constrained refinement
*wR*(*F*^2^) = 0.144	*w* = 1/[σ^2^(*F*_o_^2^) + (0.0561*P*)^2^] where *P* = (*F*_o_^2^ + 2*F*_c_^2^)/3
*S* = 0.99	(Δ/σ)_max_ = 0.001
2961 reflections	Δρ_max_ = 0.26 e Å^−^^3^
186 parameters	Δρ_min_ = −0.22 e Å^−^^3^
1 restraint	Extinction correction: none
Primary atom site location: structure-invariant direct methods	

### Special details

**Table:**

Geometry. All e.s.d.'s (except the e.s.d. in the dihedral angle between two l.s. planes) are estimated using the full covariance matrix. The cell e.s.d.'s are taken into account individually in the estimation of e.s.d.'s in distances, angles and torsion angles; correlations between e.s.d.'s in cell parameters are only used when they are defined by crystal symmetry. An approximate (isotropic) treatment of cell e.s.d.'s is used for estimating e.s.d.'s involving l.s. planes.
Refinement. Refinement of *F*^2^ against ALL reflections. The weighted *R*-factor *wR* and goodness of fit *S* are based on *F*^2^, conventional *R*-factors *R* are based on *F*, with *F* set to zero for negative *F*^2^. The threshold expression of *F*^2^ > σ(*F*^2^) is used only for calculating *R*-factors(gt) *etc*. and is not relevant to the choice of reflections for refinement. *R*-factors based on *F*^2^ are statistically about twice as large as those based on *F*, and *R*- factors based on ALL data will be even larger.

### Fractional atomic coordinates and isotropic or equivalent isotropic displacement parameters (Å^2^)

**Table:**

	*x*	*y*	*z*	*U*_iso_*/*U*_eq_	
Cl1	0.37849 (13)	0.06661 (4)	1.09548 (11)	0.0828 (4)	
O1	0.1545 (3)	0.20127 (8)	0.3759 (2)	0.0617 (6)	
O2	0.2459 (4)	0.30219 (8)	0.1123 (2)	0.0631 (6)	
H2	0.2524	0.2888	0.2008	0.095*	
N1	0.3189 (3)	0.27241 (9)	0.5512 (3)	0.0434 (6)	
N2	0.3039 (3)	0.30449 (9)	0.4155 (3)	0.0420 (6)	
C1	0.2803 (4)	0.18339 (11)	0.6672 (3)	0.0435 (7)	
C2	0.2877 (4)	0.20451 (11)	0.8154 (3)	0.0478 (7)	
H2A	0.2731	0.2432	0.8265	0.057*	
C3	0.3166 (4)	0.16914 (12)	0.9483 (4)	0.0524 (8)	
H3	0.3197	0.1834	1.0483	0.063*	
C4	0.3408 (4)	0.11202 (12)	0.9296 (4)	0.0503 (8)	
C5	0.3352 (4)	0.08999 (12)	0.7838 (4)	0.0563 (8)	
H5	0.3533	0.0514	0.7742	0.068*	
C6	0.3026 (4)	0.12538 (12)	0.6515 (3)	0.0502 (7)	
H6	0.2952	0.1106	0.5506	0.060*	
C7	0.2461 (4)	0.21899 (11)	0.5182 (3)	0.0438 (7)	
C8	0.3356 (4)	0.35814 (11)	0.4314 (3)	0.0382 (6)	
C9	0.3192 (4)	0.38770 (10)	0.2801 (3)	0.0374 (6)	
C10	0.2770 (4)	0.35866 (11)	0.1294 (3)	0.0460 (7)	
C11	0.2624 (5)	0.38790 (14)	−0.0107 (4)	0.0678 (9)	
H11	0.2341	0.3684	−0.1099	0.081*	
C12	0.2887 (5)	0.44553 (14)	−0.0070 (4)	0.0739 (10)	
H12	0.2794	0.4647	−0.1027	0.089*	
C13	0.3289 (5)	0.47455 (13)	0.1384 (4)	0.0662 (9)	
H13	0.3462	0.5136	0.1416	0.079*	
C14	0.3432 (4)	0.44635 (11)	0.2775 (4)	0.0520 (8)	
H14	0.3702	0.4667	0.3750	0.062*	
C15	0.3841 (4)	0.38928 (11)	0.5915 (3)	0.0533 (8)	
H15A	0.3203	0.3708	0.6553	0.080*	
H15B	0.3357	0.4274	0.5679	0.080*	
H15C	0.5275	0.3896	0.6543	0.080*	
H1	0.418 (3)	0.2788 (12)	0.651 (2)	0.080*	

### Atomic displacement parameters (Å^2^)

**Table:**

	*U*^11^	*U*^22^	*U*^33^	*U*^12^	*U*^13^	*U*^23^
Cl1	0.0724 (6)	0.0858 (7)	0.0841 (7)	−0.0001 (5)	0.0252 (5)	0.0420 (5)
O1	0.0699 (14)	0.0514 (12)	0.0387 (12)	−0.0031 (10)	−0.0045 (11)	−0.0021 (10)
O2	0.0910 (16)	0.0491 (13)	0.0491 (13)	−0.0110 (11)	0.0281 (13)	−0.0078 (10)
N1	0.0459 (14)	0.0396 (13)	0.0356 (13)	−0.0033 (11)	0.0068 (11)	0.0045 (11)
N2	0.0423 (14)	0.0440 (14)	0.0352 (13)	−0.0002 (11)	0.0109 (11)	0.0046 (11)
C1	0.0350 (16)	0.0387 (17)	0.0471 (18)	−0.0031 (12)	0.0064 (13)	0.0007 (13)
C2	0.0458 (18)	0.0406 (16)	0.0527 (19)	−0.0054 (13)	0.0152 (15)	0.0022 (14)
C3	0.0468 (19)	0.061 (2)	0.0483 (19)	−0.0079 (15)	0.0176 (15)	0.0026 (15)
C4	0.0351 (16)	0.055 (2)	0.054 (2)	−0.0018 (14)	0.0112 (14)	0.0203 (15)
C5	0.0476 (19)	0.0370 (17)	0.075 (2)	−0.0006 (13)	0.0151 (17)	0.0109 (16)
C6	0.0485 (18)	0.0444 (18)	0.0493 (18)	−0.0050 (13)	0.0109 (14)	−0.0002 (14)
C7	0.0409 (16)	0.0411 (17)	0.0418 (17)	0.0016 (13)	0.0086 (14)	0.0030 (13)
C8	0.0313 (15)	0.0397 (16)	0.0386 (16)	0.0016 (12)	0.0088 (12)	−0.0008 (12)
C9	0.0321 (14)	0.0415 (16)	0.0361 (15)	0.0031 (12)	0.0109 (12)	0.0035 (12)
C10	0.0499 (18)	0.0441 (18)	0.0441 (17)	0.0026 (13)	0.0190 (14)	0.0020 (14)
C11	0.089 (3)	0.073 (2)	0.0403 (19)	−0.0044 (19)	0.0249 (17)	0.0029 (16)
C12	0.093 (3)	0.069 (2)	0.055 (2)	−0.005 (2)	0.0241 (19)	0.0206 (18)
C13	0.080 (2)	0.050 (2)	0.062 (2)	−0.0014 (17)	0.0216 (19)	0.0116 (17)
C14	0.061 (2)	0.0421 (18)	0.0490 (18)	0.0039 (14)	0.0184 (15)	0.0048 (14)
C15	0.070 (2)	0.0473 (18)	0.0411 (17)	0.0005 (15)	0.0204 (15)	−0.0006 (14)

### Geometric parameters (Å, °)

**Table:**

Cl1—C4	1.736 (3)	C5—H5	0.93
O1—C7	1.225 (3)	C6—H6	0.93
O2—C10	1.353 (3)	C8—C9	1.459 (3)
O2—H2	0.82	C8—C15	1.493 (3)
N1—C7	1.355 (3)	C9—C14	1.400 (3)
N1—N2	1.375 (3)	C9—C10	1.406 (3)
N1—H1	0.896 (10)	C10—C11	1.373 (4)
N2—C8	1.287 (3)	C11—C12	1.375 (4)
C1—C2	1.370 (4)	C11—H11	0.93
C1—C6	1.395 (4)	C12—C13	1.369 (4)
C1—C7	1.486 (4)	C12—H12	0.93
C2—C3	1.377 (4)	C13—C14	1.354 (4)
C2—H2A	0.93	C13—H13	0.93
C3—C4	1.381 (4)	C14—H14	0.93
C3—H3	0.93	C15—H15A	0.96
C4—C5	1.363 (4)	C15—H15B	0.96
C5—C6	1.369 (4)	C15—H15C	0.96
			
C10—O2—H2	109.5	N2—C8—C15	123.5 (2)
C7—N1—N2	116.2 (2)	C9—C8—C15	121.1 (2)
C7—N1—H1	117 (2)	C14—C9—C10	116.8 (2)
N2—N1—H1	120 (2)	C14—C9—C8	121.6 (2)
C8—N2—N1	120.1 (2)	C10—C9—C8	121.6 (2)
C2—C1—C6	119.3 (3)	O2—C10—C11	116.7 (3)
C2—C1—C7	123.5 (2)	O2—C10—C9	123.3 (2)
C6—C1—C7	117.1 (3)	C11—C10—C9	120.0 (3)
C1—C2—C3	120.8 (3)	C10—C11—C12	121.2 (3)
C1—C2—H2A	119.6	C10—C11—H11	119.4
C3—C2—H2A	119.6	C12—C11—H11	119.4
C2—C3—C4	118.6 (3)	C13—C12—C11	119.6 (3)
C2—C3—H3	120.7	C13—C12—H12	120.2
C4—C3—H3	120.7	C11—C12—H12	120.2
C5—C4—C3	121.8 (3)	C14—C13—C12	119.9 (3)
C5—C4—Cl1	118.7 (2)	C14—C13—H13	120.1
C3—C4—Cl1	119.5 (2)	C12—C13—H13	120.1
C4—C5—C6	119.1 (3)	C13—C14—C9	122.5 (3)
C4—C5—H5	120.4	C13—C14—H14	118.7
C6—C5—H5	120.4	C9—C14—H14	118.7
C5—C6—C1	120.4 (3)	C8—C15—H15A	109.5
C5—C6—H6	119.8	C8—C15—H15B	109.5
C1—C6—H6	119.8	H15A—C15—H15B	109.5
O1—C7—N1	122.7 (2)	C8—C15—H15C	109.5
O1—C7—C1	121.9 (2)	H15A—C15—H15C	109.5
N1—C7—C1	115.4 (2)	H15B—C15—H15C	109.5
N2—C8—C9	115.3 (2)		

### Hydrogen-bond geometry (Å, °)

**Table:**

*D*—H···*A*	*D*—H	H···*A*	*D*···*A*	*D*—H···*A*
O2—H2···N2	0.82	1.80	2.513 (3)	145
N1—H1···O1^i^	0.90 (1)	2.074 (11)	2.968 (3)	176 (3)

Symmetry codes: (i) *x*+1/2, −*y*+1/2, *z*+1/2.
